# Patients’ Expectations for App-Based Therapy in Knee Osteoarthritis: User-Centered Design Approach

**DOI:** 10.2196/64607

**Published:** 2025-05-15

**Authors:** Pika Krištof Mirt, Karmen Erjavec, Sabina Krsnik, Petra Kotnik, Hussein Mohsen

**Affiliations:** 1 General Hospital Novo Mesto, Novo Mesto, Slovenia; 2 Department of Physiotherapy, Faculty of Health Sciences, University of Novo Mesto, Na Loko 2, Novo Mesto, 8000, Slovenia, 386 040869839; 3 Artros Medical Center, Ljubljana, Slovenia

**Keywords:** knee osteoarthritis, experience, opinion, perspective, mHealth, app, user-centered design, patient engagement, preference, physiotherapy, exercises, motivation, functional requirements, barriers and benefits, digital health, chronic disease management, joint, arthritis, osteoarthritis, rheumatology

## Abstract

**Background:**

Knee osteoarthritis (KOA) requires long-term treatment that faces significant barriers, including inadequate physiotherapy services, especially in Slovenia and comparable European countries. Mobile health apps offer a promising solution to improve accessibility and adherence to KOA treatment.

**Objectives:**

This study aimed to identify expectations of patients with KOA for app-based therapy, determine the functional requirements, and assess the main barriers and benefits of using mobile apps for KOA management. It also examined these factors about demographic data (gender, age, and education level) and motivation to perform knee exercises.

**Methods:**

A mixed methods approach was used, integrating quantitative data from a structured questionnaire and qualitative data from in-depth interviews. The purposive sample comprised 82 patients with symptomatic KOA graded 1‐3 on the Kellgren-Lawrence scale, excluding those with cognitive impairments, wheelchair dependency, significant comorbidities, or language barriers.

**Results:**

The analysis revealed that 53.7% (44/82) of patients preferred smartphones, while 40.2% (33/82) favored PCs for remote KOA management, citing accessibility and convenience. Exercise videos received the highest rating (µ=9.45), followed by goal setting and tracking (µ=8.95) and regular e-messages (µ=8.83). Telephone consultations with physiotherapists were also highly valued (µ=8.41). Significant differences were observed in the perceived importance of key disease information (*F*_9_=2.077; *P*=.04) and exercise videos (*F*_9_=2.788; *P*=.05) based on motivation levels but not by gender, age, or education. Perceptions of the appropriate duration of physical activity varied with motivation levels (*F*_9_=2.490; *P*=.02) but not with demographic factors. Men rated ease of use (4.93 vs 4.71; *F*_1_=3.961; *P*=.05) and the clarity of the exercise flow display higher than women. The most significant barrier was inaccurate disease information (µ=3.96), with notable differences across age groups. Younger participants (younger than 40 years) and those aged 51‐60 years expressed concerns about time management and information accuracy. Patients highlighted the ability to rewatch exercises as a key app feature, while time efficiency and improved access to physiotherapists were highly valued for convenience. Enhanced communication and accurate information were essential for building trust and ensuring effective treatment.

**Conclusions:**

Mobile health apps for KOA management should be designed with a user-centered approach, prioritizing accessibility, motivation, and effective communication. Key functionalities include high-quality exercise videos, goal setting, symptom tracking, and regular electronic reminders. Mitigating user-reported barriers and integrating age-specific adaptations can enhance adherence and therapeutic outcomes. The findings highlight the potential of mobile health technologies to optimize KOA self-management and improve patient quality of life, particularly in health care systems with limited physiotherapy accessibility, such as those in Slovenia.

## Introduction

Knee osteoarthritis (KOA) is a prevalent degenerative joint disease affecting 16% of individuals aged 15+ years and 22.9% of those aged 40+ years [1]. As a leading musculoskeletal disorder [2], it poses a growing economic and health burden [3-5], necessitating effective management strategies to mitigate symptoms and improve patient outcomes.

Exercise, self-management, and patient education are essential for KOA management, as recommended by the Osteoarthritis Research Society International and the American College of Rheumatology [6-9]. However, barriers such as misinformation, low self-efficacy, limited physiotherapy access, and restrictive care pathways hinder their effectiveness [10-16]. In Slovenia, the shortage of physiotherapists (50‐70 per 100,000 inhabitants) leads to prolonged waiting times, a challenge also reported in Eastern and Southern Europe, as well as in the United Kingdom, Canada, and Australia, where delayed access to physiotherapy negatively impacts treatment outcomes [17,18]. Given KOA’s significant health care, economic, and social challenges, efforts are focused on developing effective, accessible treatments. As defined by the World Health Organization, mobile health technology includes medical practices supported by mobile devices [19]. Mobile health apps offer scalable solutions by delivering behavioral interventions, facilitating remote monitoring, and providing access to educational resources and clinical guidelines. Health apps support patients in adhering to exercise regimens, managing symptoms, and making informed health decisions, effectively bridging the gap between patients and health care providers, thus improving KOA management and outcomes [10‐14].

The use of digital health technologies for managing KOA is gaining attention due to its cost-effectiveness and accessibility. Systematic reviews and meta-analyses have found that digital self-management interventions for managing KOA—delivered through mobile apps, internet platforms, telephone, audio, and video—lead to small to moderate improvements in pain and function, with effects lasting up to 1 year [20]. McHugh et al [21] found that remote exercise programs are more effective when they include study-initiated follow-up and behavior change counseling. Choi et al [22] emphasized the need for mobile health technologies to support self-management and collaborative decision-making. Shah et al [23] confirmed that digital interventions, including cognitive behavioral therapy and tools that facilitate patient-clinician communication, are as effective as traditional methods for managing KOA and highlighted the importance of integrating patient preferences and contextual factors, such as education level, into the design of digital health interventions, which are often overlooked in current research.

Previous qualitative studies have demonstrated that gender and age influence user engagement and preferences. Women often value comprehensive health information and interactive support features, while men prioritize ease of use and efficiency [24]. Younger users prefer advanced features and detailed tracking, whereas older users may face challenges with digital literacy, favoring simpler interfaces with larger text and intuitive navigation [25,26]. Past studies have also indicated that patients with higher levels of education are more willing to engage with advanced app features and provide detailed feedback, favoring functionalities such as data tracking and interactive educational resources [24,27]. Existing qualitative research has established motivation as a critical factor influencing app-based therapy adoption and sustained use. McHugh et al [21] demonstrated that follow-up and behavior change counseling interventions are more effective, emphasizing motivation’s role in app engagement.

Although mobile apps hold promise for KOA management, their adoption remains low. Most users discontinue use within 90 days due to suboptimal development methods that fail to support regular communication and patient motivation [28]. Existing mobile apps for KOA management face limitations, including suboptimal user engagement, insufficient personalization, inadequate integration with health care providers, and poor long-term adherence. This highlights the need for personalized goal setting, real-time feedback, automated phone call reminders, and regular clinician-patient communication to enhance engagement, adherence, and clinical effectiveness. In this context, high-quality exercise videos play a crucial role by adhering to evidence-based physiotherapy principles, providing clear demonstrations, precise instructions, appropriate pacing, and modifications for different skill levels, and ensuring safety, accuracy, and effectiveness in guided exercises [28-31]. Studies advocate a co-design approach involving end users in developing, testing, and refining interventions to improve patient satisfaction and app effectiveness [28-31]. This study investigated the user-centered app design process with patients with KOA to develop a more effective app-based therapy.

This study addresses a critical gap in the co-design process for developing a KOA mobile app by using a mixed methods approach within the context of an underresourced health care system, such as Slovenia. While mixed methods research has been used in evaluating digital health interventions, previous studies have primarily focused on other rehabilitation and assistive technologies domains [32-34], with limited application to KOA management. This study aimed to examine the expectations of app-based therapy of patients with KOA, identify key functional requirements (eg, personalized goal setting, real-time feedback, and automated reminders), and assess barriers and benefits of mobile app use for KOA management. Furthermore, it analyzed these factors concerning demographic characteristics (gender, age, and education) and motivation levels. The study tested the hypothesis that perceptions of functional requirements, app usefulness, barriers, and benefits vary significantly based on demographic characteristics (age, gender, and education) and motivation levels.

## Methods

### Study Design

The study used a structured co-design approach based on participatory design principles [35], ensuring active stakeholder involvement. Using the Double Diamond framework, we conducted qualitative data collection to identify user needs (Discover), synthesized insights to define core functionalities (Define), iteratively developed prototypes with patients’ feedback (Develop), and assessed usability and feasibility (Deliver) [35].

This study adopted a sequential explanatory mixed methods design, integrating quantitative and qualitative data to understand the research problem comprehensively. Integration occurred at multiple stages, including planning (study design), sampling (purposive selection based on survey results), data collection (qualitative interviews following quantitative analysis), data analysis (triangulation of qualitative themes with quantitative findings), and interpretation (integrated reporting for contextual depth). The quantitative analysis identified key trends and patterns, which then informed the development of qualitative interviews, allowing for an in-depth exploration of the emerging themes. A triangulation approach was applied to ensure systematic integration, mapping qualitative themes onto quantitative results to assess convergence, complementarity, and divergence. This method enabled a deeper contextualization of statistical findings, strengthening the study’s validity, interpretability, and overall rigor [32-34].

### Ethical Considerations

The National Committee on Medical Ethics of the Republic of Slovenia (no. 0120-471/2023-2711-4) granted ethical approval for this study. Informed consent was obtained from all participants before their inclusion in the study. Participants were thoroughly briefed on the research aims, their right to discontinue participation at any stage without penalty, and the voluntary nature of their involvement. To safeguard privacy and confidentiality, all data were anonymized before analysis, with no personally identifiable information being stored or disclosed. Data were securely stored on password-protected devices, accessible exclusively to research team members. No financial or material incentives were provided, as the study was designed to impose a minimal burden and relied solely on voluntary participation.

### Quantitative Survey

A quantitative survey was conducted between March and June 2024 among patients with KOA recruited through purposive sampling to ensure a representative distribution of demographic characteristics and disease experiences. The sample size (n=82) was determined using power calculations, ensuring adequate statistical precision and validity. Participants were recruited after specialist examinations at Artros, Slovenia’s leading orthopedic clinic. They met the inclusion criteria of having symptomatic KOA graded 1‐3 on the Kellgren-Lawrence Scale. Patients with cognitive impairment, wheelchair dependency, comorbidities, or limited language or digital skills were excluded. All participants provided informed consent and completed a 10-minute questionnaire, resulting in 82 completed surveys.

The instrument was developed based on measures or findings from previous studies [22-27] and refined for the Slovenian context through interviews with 5 experts, including academics and health professionals. It was piloted with 25 professionals (physiotherapists, rehabilitation specialists, and orthopedic experts) and patients to ensure clarity and relevance, incorporating various question types and scales for comprehensive data collection.

The survey tool was a structured questionnaire comprising 38 items designed to assess demographic characteristics, motivation, expectations, usability, barriers, and benefits related to a web-based application for KOA rehabilitation. It included multiple-choice questions, Likert scale ratings (1-10), and binary response options. The questionnaire consisted of 6 main sections: demographic data covering gender, age, education, and occupation; motivation to perform knee exercises, assessed using a 10-point Likert scale ranging from 1 (not motivated at all) to 10 (extremely motivated) and categorized as low (1-6) and high (7-10) based on established thresholds; expectations from the digital app (9 items), evaluating the importance of various app features such as educational content, goal setting, video demonstrations, reminders, and communication with physiotherapists; usability perception (6 items), measuring ease of use, navigation, and information clarity; potential barriers (10 items), identifying challenges such as technical difficulties, lack of web access, and low motivation; and perceived benefits (6 items), assessing factors such as time savings, improved motivation, and enhanced physiotherapist interaction (Multimedia Appendix 1). The questionnaire was self-administered in paper format within a health care setting, ensuring consistency in data collection while allowing respondents to complete it independently in a confidential environment. The questionnaire demonstrated high internal consistency, with a Cronbach alpha value of 0.85, indicating robust reliability.

### Qualitative Study

In-depth interviews were conducted to understand the patient’s needs, functional requirements, and esthetic preferences for a mobile KOA management app and identify barriers and facilitators to its use. After completing the quantitative survey administered by questionnaire (n=82 patients; Table 1), qualitative interviews were conducted with all selected participants, ensuring a comprehensive exploration of their experiences and perspectives.

**Table 1.** Demographic characteristics (n=82).

Attribute	Share of total respondents, n (%)
Sex	
Male	27 (32.9)
Female	55 (67.1)
Age (years)	
31‐40	2 (2.4)
41‐50	15 (18.3)
51‐60	25 (30.5)
61‐70	23 (28.0)
71‐80	17 (20.7)
Highest education level	
Secondary school or less	54 (65.9)
Bachelor’s degree	24 (29.3)
Master’s degree/specialization	3 (4.9)
Doctoral degree	N/A^a^

a N/A: not applicable.

A semistructured interview guide, developed by reviewing existing literature [24-31] and expert consultation, provided a structured yet flexible framework for in-depth discussions. Following the initial interviews, the guide was carefully reviewed and refined, with minor adjustments made to improve clarity and relevance in response to emerging themes. Patients were asked to provide detailed insights into their expectations, needs, functional requirements, and esthetic preferences for a mobile KOA management app. In addition, they shared their views on barriers to app usage and potential facilitators. Trained researchers with expertise in qualitative health research conducted interviews. These interviews took place face-to-face in confidential and comfortable settings, such as quiet rooms in health care facilities or other mutually agreed locations, ensuring participant comfort and open communication. Each interview lasts between 45 and 90 minutes. All interviews were audio-recorded with the participant’s informed consent and transcribed verbatim.

### Data Management and Analysis

The quantitative analysis was conducted using descriptive statistics to analyze mean (µ) values and the normality of data distribution was assessed using the Shapiro-Wilk test, which confirmed a normal distribution (*P*>.05). Consequently, parametric tests were used for further analysis. Differences between groups were evaluated using the independent samples *t* test and ANOVA, with statistical significance set at *P*<.05. All data analyses were performed using SPSS (version 25.0; IBM Corp).

For the qualitative component, we conducted the thematic analysis using NVivo (version 12; QSR International) software to facilitate systematic coding and organization of data. Two researchers (KE and PK) independently coded the transcripts, ensuring intercoder reliability and reducing potential bias. We applied an inductive coding approach, where codes were derived directly from the data and categorized into broader themes. We used an iterative coding process to address overrepresentation and potential errors, cross-checked themes against the dataset, and resolved discrepancies through researcher discussions and consensus-building. Themes were identified through pattern recognition, with recurrent concepts reviewed for coherence and alignment with the research objectives. Differences in interpretation were resolved through researcher consensus, with a third researcher consulted when necessary to ensure analytical rigor and objectivity.

## Results

### Overview

The results will be presented in thematic sections, explaining the quantitative findings with qualitative data. The study included 82 participants, with 67.1% (55) female and 32.9% (27) male respondents. The majority were aged 51‐70 years (70, 58.5%), while smaller proportions were aged 31‐40 years (2, 2.4%) and 71‐80 years (17, 20.7%). Regarding education, 65.9% (54) had secondary school or less, 29.3% (24) held a bachelor’s degree, and 4.9% (4) had a master’s degree or specialization, with no participants holding a doctoral degree (Table 1).

### Preferred Device for Remote KOA Management

Most respondents preferred performing KOA management via smartphone (44, 53.7%) or PC (33, 40.2%) due to their accessibility, convenience, and technical possibilities, as the in-depth interviews revealed. Smartphones offer quick and easy access to apps anywhere and anytime, increasing the likelihood of regular exercise. With their larger screens, PCs offer a better overview of exercise videos and instructions. Both devices support high-quality video content and easy web connectivity, which is essential for monitoring progress and communicating with physiotherapists. According to patient 1, “With the phone, I could exercise anywhere, while with the computer, I could have a better overview of the instructions and exercises.… So, I could combine them depending on the situation.”

### Importance of the Functional Requirements of Mobile Apps for KOA Management

The results showed that the importance of functional requirements for mobile apps managing KOA did not significantly vary between genders, age groups, or educational levels. Table 2 demonstrates that differences in the importance of functional requirements for KOA management mobile apps are significant among various motivation levels for only 2 features: “key information about the disease” and “videos with different exercises.” Patients with higher motivation levels expressed a greater desire for comprehensive information about their condition, including disease details, treatment strategies, and healthy lifestyle tips. Specifically, those who reported higher motivation also placed greater value on having key information about the disease included in the app, as indicated by the significant differences in mean motivation levels (*F*_9_=2.077; *P*=.04). Qualitative analysis also confirmed that highly motivated patients seek detailed information to support their KOA management. A typical statement was from patient 3: “Having detailed information about my condition and access to exercise videos could motivate me to stay on track with my therapy.” Similarly, the data indicated that highly motivated patients emphasized the availability of videos with different exercises for knee strengthening. These patients rated the importance of exercise videos significantly higher than those with lower motivation levels (*F*_9_=2.788; *P*=.01). This suggests that motivated patients are more likely to engage with an app that provides clear, visual guidance through exercise videos, enhancing their ability to perform exercises correctly and consistently. Patient 4 stated: “The exercise videos in the app will help me to understand how to do the exercises correctly and motivate me to keep going.”

**Figure 1.**
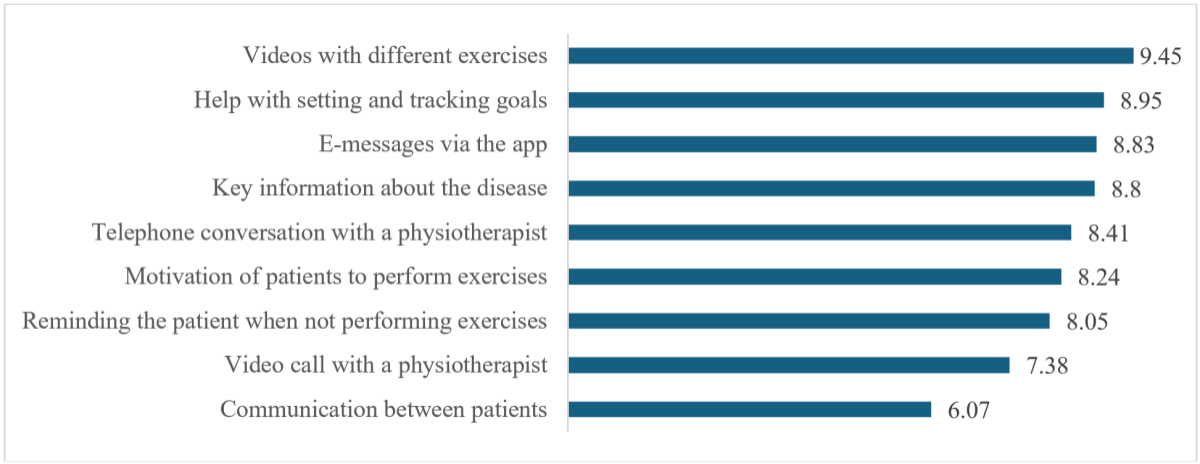
Importance of the functional requirements of mobile apps for knee osteoarthritis management.

Figure 1 illustrates the importance ratings of various functional requirements of mobile apps for KOA management, measured on a Likert scale from 1 (not important) to 10 (extremely important), reflecting participant preferences. Analysis of the in-depth interviews showed that patients rated the importance of these features based on their practical needs and preferences in managing their condition. The highest-rated function, videos with different exercises (µ=9.45), reflected the patients’ need for clear, visual guidance to ensure that the exercises were performed correctly, as the analysis of patient statements revealed. Setting and tracking goals (µ=8.95) was also rated highly, as patients considered it essential for monitoring their progress and staying motivated. Regular e-messages via the app (mean rating: 8.83) and essential information about the disease (µ=8.80) were identified as crucial for keeping patients informed and involved in their treatment plans. Direct communication with health care providers was deemed important, as evidenced by the high rating of phone calls with a physiotherapist (µ=8.41), which suggests that patients value personalized advice and reassurance from professionals. Features that support treatment adherence, such as motivation to perform exercises (µ=8.24) and reminders when exercises are not performed (µ=8.05), were considered critical for maintaining a consistent exercise program. Although video calls with a physiotherapist (µ=7.38) and communication between patients (µ=6.07) were rated lower, they were still important for some patients, especially those seeking more direct interaction or peer support.

**Table 2.** Differences in the importance of the functional requirements of mobile apps for knee osteoarthritis management regarding the level of motivation (ANOVA)^a^.

Level of motivation		Sample size, n	µ^b^	σ^c^	*F* test (*df*)^d^	*P* value
Key information about the disease					2.07 (9)	.04
1		1	5.00	0.000		
2		2	6.50	4.950		
3		4	10.00	0.000		
4		2	8.00	2.828		
5		9	8.67	2.646		
6		8	8.00	3.071		
7		9	9.33	1.414		
8		22	8.05	2.420		
9		9	9.67	0.500		
10		16	9.88	0.500		
Videos with different exercises					2.788 (9)	.007
1		1	7.00	0.000		
2		2	6.50	4.950		
3		4	10.00	0.000		
4		2	9.00	1.414		
5		9	9.00	2.000		
6		8	9.63	1.061		
7		9	9.67	0.707		
8		22	9.32	1.041		
9		9	9.67	0.500		
10		16	10.00	0.000		

a All motivation measures in this and subsequent tables use a scale from 1 (lowest) to 10 (highest).

b Mean score of importance ratings for each functional requirement.

c SD values.

d ANOVA test statistic.

### Appropriate Amount of Time to Exercise

Table 3 shows participants’ preferences regarding the reasonable duration for app-based exercises, with response options of up to 15 minutes, 16‐30 minutes, and 31‐45 minutes. The results indicate that opinions on the appropriate exercise duration did not significantly differ based on gender, age, or educational level. This suggests a consensus among participants on the time they are willing to dedicate to physical activity within the mobile app. The analysis of the in-depth interviews revealed that a consensus between different demographic groups regarding the recommended duration of physical activity can be attributed to shared experience. Patients with KOA often experience similar symptoms, such as pain and stiffness, leading to a shared understanding of the benefits of exercise. Patient 5 said, “I know many people with this condition and can say that we all feel the same relief from exercise, regardless of our positions, so the recommended duration works for everyone.”

As shown in Table 3, there was a statistically significant difference in opinion regarding the level of motivation based on the appropriate amount of time to exercise (*F*_9_=2.490; *P*=.0.15). Patients with higher motivation levels (ratings of 7‐10) favored longer exercise durations (mean ratings from 2.18 to 2.31) than those with lower motivation levels (ratings of 1‐6, mean ratings from 1.00 to 2.00). A typical patient statement in this regard was from patient 6: “I find that longer exercise sessions could help me feel more accomplished and keep me motivated to stay consistent with my routine.”

**Table 3.** Differences in opinions of the appropriate amount of time to exercise regarding the level of motivation (ANOVA).

Category		Sample size, n	µ^a^	σ^b^	*F* test (*df*)^c^	*P* value
Depending on the level of motivation					2.490 (9)	.02
1		1	2.00	0.000		
2		2	2.00	1.000		
3		4	2.50	0.289		
4		2	1.00	0.000		
5		9	1.33	0.167		
6		8	1.88	0.295		
7		9	2.22	0.278		
8		22	2.18	0.125		
9		9	2.22	0.222		
10		16	2.31	0.151		

a Mean score of importance ratings for each functional requirement.

b SD values.

c ANOVA test statistic.

### Usefulness of Mobile Exercise Apps for KOA Management

Table 4 indicates statistically significant differences in the assessment of the usefulness of mobile apps based on gender, measured on a 1‐5 Likert scale (1=I do not agree at all, 5=I totally agree). According to the data, men generally rated KOA apps better than women in terms of overall ease of use, clarity of system information, and general friendliness. They also had significantly higher ratings than women for ease of use (µ=4.93 vs µ=4.71; *F*_1_=3.961; *P*=.05), clarity of the exercise process display higher (µ=4.93 vs µ=4.71; *F*_1_=3.961; *P*=.05), and ease of navigation on the tab (µ=4.89 vs µ=4.64; *F*_1_=3.961; *P*=.04). In addition, men rated instructions on performing exercises higher (µ=4.93, σ=0.267) than women (µ=4.69; *F*_1_=4.318; *P*=.04). Their ratings were also higher than women’s ratings regarding providing help on how to perform exercises correctly (µ=4.93 vs µ=4.67; *F*_1_=4.653; *P*=.03) and ease of communication with the physiotherapist (µ=4.78 vs µ=4.49; *F*_1_=4.196; *P*=.04). These results show that, on average, men have higher expectations of user-friendliness, clarity of system information, and ease of app use than women.

**Table 4.** Differences in opinions of the usefulness of mobile exercise apps based on gender (*t* test).

	Sample size, n	µ^a^	Σ^b^	*F* test (*df*)^c^	*P* value
Ease of use				3.961 (1)	.05
Male	27	4.93	0.267		
Female	55	4.71	0.533		
A clear display of the training process				3.961 (1)	.05
Male	27	4.93	0.267		
Female	55	4.71	0.533		
Easy tab navigation				3.961 (1)	.04
Male	27	4.89	0.320		
Female	55	4.64	0.589		
Instructions for carrying out the exercises				4.318 (1)	.04
Male	27	4.93	0.267		
Female	55	4.69	0.540		
Help with the correct execution of exercises				4.653 (1)	.03
Male	27	4.93	0.267		
Female	55	4.67	0.579		
Facilitated communication with the physiotherapist				4.196 (1)	.04
Male	27	4.78	0.424		
Female	55	4.49	0.663		

a Mean score of importance ratings for each functional requirement..

b SD values.

c ANOVA test statistic.

The analysis of in-depth interviews revealed that gender differences in the perceived usefulness of mobile apps were attributable to distinct priorities and user experiences between men and women. Men rated ease of use, clarity of system information, and overall usability higher than women, indicating a preference for simple, efficient interfaces and clear, structured data. For example, patient 9, a male, stated, “I find it very important that the app is easy to use so that I can get to my exercises quickly and without problems,” making it clear that he valued functionality and efficiency. Similarly, the qualitative analysis showed that men placed more value on a clear display of exercise progress and easy navigation of the tabs, as indicated by the feedback: “A clear display of my progress will help me stay motivated and keep track of my workouts” (Patient 11). This suggests that men prefer features that improve their ability to independently track and manage their workout routines. Furthermore, the importance placed on detailed instructions and help in performing exercises correctly, with statements such as “Exact instructions and assist could help with exercises ensure that I perform them correctly and safely” (Patient 12), indicates a preference for precision and correctness in their exercise plans. Finally, the higher rating of facilitated communication with physiotherapists, supported by statements such as “Being able to communicate with the physiotherapist via the app is very helpful to get quick feedback and advice” (Patient 13), underscores the importance of direct and immediate support.

Table 5 shows an ANOVA analysis examining differences in perceptions of mobile app usefulness based on motivation levels (ranging from 1 to 10). The analysis revealed statistically significant differences for only 2 specific functionalities, with “Ease of Use” being the only factor reaching statistical significance: ease of use (*F*_9_=2.397; *P*=.02) and good organization of information (*F*_9_=2.358; *P*=.02). Highly motivated patients (rating 10) rated user-friendliness the highest (µ=5.00), while those with lower motivation (rating 1) rated it lower (µ=4.00). Good organization of information was particularly important for those with very high or low motivation, with ratings of 3, 4, and 10 yielding a mean score of 5.00, while those with medium motivation (rated 7) assigned a lower score of 4.11. The in-depth interviews revealed that the differences in opinions about the ease of use and organization of the information in the mobile app were due to users’ different needs and expectations based on their motivation levels. Highly motivated individuals desired efficient and straightforward tools to maintain their exercise routine, as in patient 14’s statement: “The app needs to be very user-friendly because, as it now says, it should make me engage with my exercises and stay consistent.” They would like an intuitive user interface to maximize their productivity and stick to their programs. Conversely, both less motivated and highly motivated users placed a high value on well-organized information, as clarity would help them focus and reduce cognitive load, improving comprehension and engagement. Patient 8 illustrated this statement: “When all the information is clear, I can focus better and know what I need to do next.”

**Table 5.** Differences in opinions of the usefulness of mobile apps based on the level of motivation (ANOVA).

Level of motivation	Sample size, n	µ^a^	Σ^b^	*F* test (*df*)^c^	*P* value
Ease of use				2.397 (9)	.02
1	1	4.00	0.000		
2	2	4.00	1.414		
3	4	4.75	0.500		
4	2	5.00	0.000		
5	9	4.56	0.726		
6	8	4.88	0.354		
7	9	4.67	0.500		
8	22	4.91	0.294		
9	9	4.56	0.527		
10	16	5.00	0.000		
Good organization of information in the app				2.358 (9)	.02
1	1	4.00	0.000		
2	2	4.00	1.414		
3	4	5.00	0.000		
4	2	5.00	0.000		
5	9	4.67	0.707		
6	8	4.88	0.354		
7	9	4.11	1.364		
8	22	4.91	0.294		
9	9	4.67	0.500		
10	16	5.00	0.000		

a Mean score of importance ratings for each functional requirement.

b SD values.

c ANOVA test statistic.

### Barriers to Using a Mobile App for KOA Management

**Figure 2.**
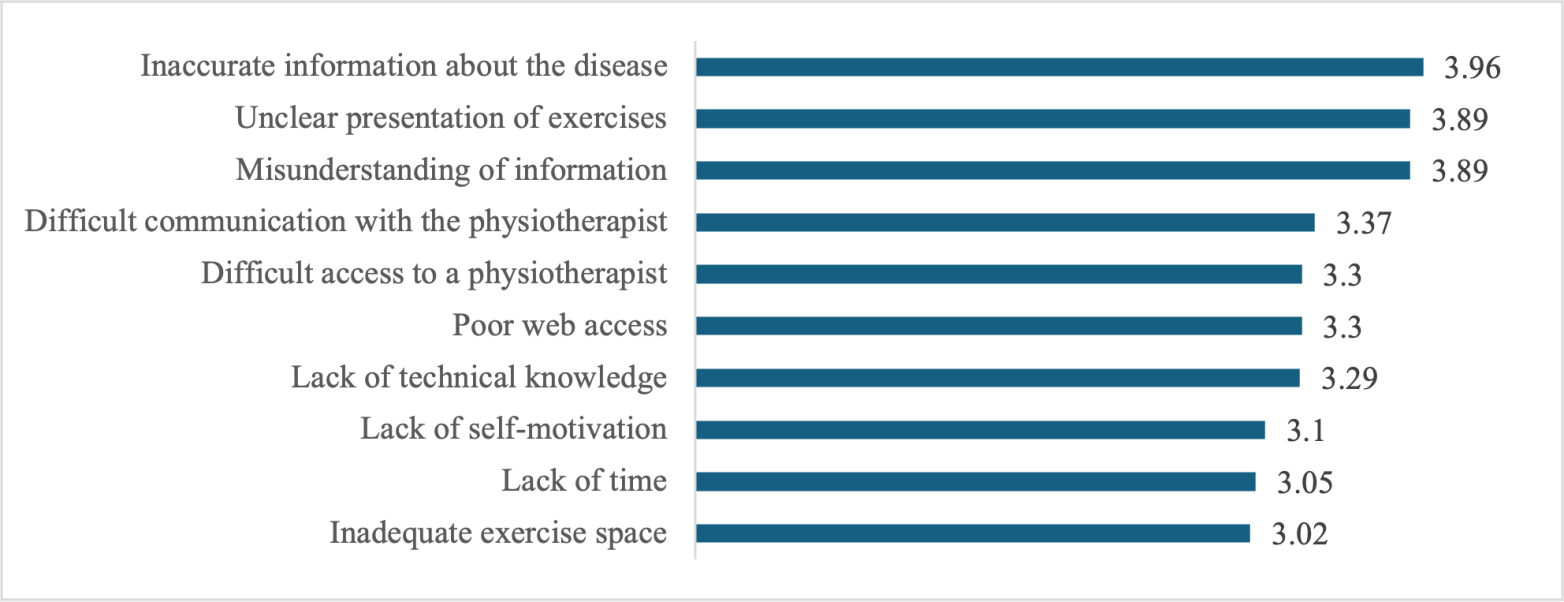
Barriers to using a mobile app for knee osteoarthritis management (n=82).

Figure 2 illustrates the barriers to using a mobile app to manage KOA, measured on a 1‐5 Likert scale (1=I do not agree at all, 5=I totally agree). The highest-rated barrier was inaccurate information about the disease (3.96), closely followed by unclear presentation of exercises (3.89) and misunderstanding of information (3.89). Difficult communication with the physiotherapist (3.37) and access to a physiotherapist (3.3) were also important barriers. Poor web access (3.3) and lack of technical knowledge (3.29) reflected technology-related challenges. In addition, lack of self-motivation (3.1), lack of time (3.05), and inadequate training space (3.02) were moderate barriers.

Table 6 shows the results of an ANOVA analysis examining differences in the perceived barriers to using a mobile app for KOA management across different age groups. Participants aged 51‐60 years reported the highest barrier scores for inaccurate information about the disease (µ=4.40), with younger participants younger than 40 years also expressing significant concern (µ=4.00). Similarly, lack of time was identified as an important barrier by participants younger than 40 years (µ=4.50) and those aged 51‐60 years (µ=3.72). Motivation-related challenges were most pronounced among the youngest age group (µ=4.50, σ=0.707), while the unclear presentation of exercises was a key barrier for those younger than 40 years (µ=4.50, σ=0.707) and those aged 51‐60 years (µ=4.36, σ=0.810). Participants aged 51‐60 years exhibited a relatively low SD, suggesting greater consensus in their perceptions. In contrast, younger participants demonstrated higher SD values, indicating greater response variability, particularly regarding motivation and clarity of exercise instructions.

The in-depth interviews revealed that the barriers to KOA management via mobile apps stem from individual needs, lifestyle restrictions, and expectations of the app. Patients emphasized the need for reliable information: “You know, accurate information is the most important thing for me to trust the app” (Patient 4). Time constraints were important, especially for younger patients, as patient 22 explained: “I find it difficult to fit exercise into my busy schedule.” Motivation was also an important issue, requiring clear goals and support, as noted by patient 23: “Without clear goals and support, it’s difficult to stay motivated. And without motivation, you can’t do it, so it’s a big problem.” In addition, the need for clear exercise instructions was crucial, as patient 24 emphasized: “I need clear instructions to make sure I’m doing the exercises correctly.”

**Table 6.** Differences in barriers to using apps based on age (ANOVA).

Age (years)	Sample size, n	µ^a^	σ^b^	*F* test^c^	*P* value
Inaccurate information about the disease				2.774 (4)	.03
<40	2	4.00	1.414		
41‐50	15	3.67	1.397		
51‐60	25	4.40	0.707		
61‐70	23	4.17	1.072		
71‐80	17	3.29	1.490		
Lack of time				4.107 (4)	.005
<40	2	4.50	0.707		
41‐50	15	2.93	1.486		
51‐60	25	3.72	0.891		
61‐70	23	2.65	1.335		
71‐80	17	2.53	1.179		
Lack of motivation				2.661 (4)	.04
<40	2	4.50	0.707		
41‐50	15	3.00	1.254		
51‐60	25	3.56	1.215		
61‐70	23	3.09	0.561		
71‐80	17	2.35	1.272		
Unclear presentation of exercises				2.515 (4)	.048
<40	2	4.50	0.707		
41‐50	15	3.47	1.246		
51‐60	25	4.36	0.810		
61‐70	23	3.96	1.147		
71‐80	17	3.41	1.417		

a Mean score of importance ratings for each functional requirement.

b SD values.

c ANOVA test statistic.

### Benefits of Using a Mobile App for KOA Management to Manage KOA

**Figure 3.**
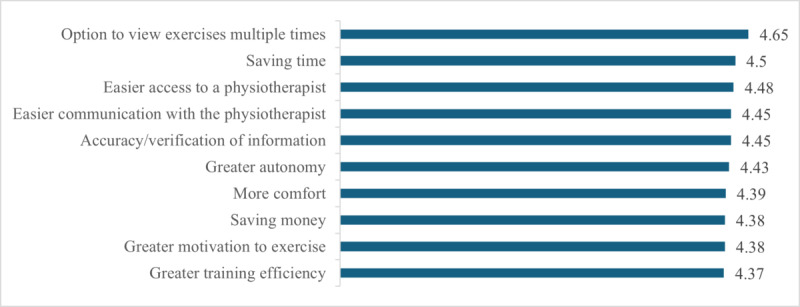
Benefits of using a mobile app to manage knee osteoarthritis (N=82).

Figure 3 illustrates the benefits of using a mobile app to manage KOA, measured on a 1‐5 Likert scale (1=I do not agree at all, 5=I totally agree). The highest-rated benefit was the ability to view exercises multiple times (µ=4.65), which indicates how important clear, repeatable instructions are to users. Saving time (µ=4.50) and easier access to a physiotherapist (µ=4.48) were also rated highly, reflecting the importance of convenience and accessibility. Improved communication with the physiotherapist (µ=4.45) and the accuracy of information provided (µ=4.45) were critical to building trust and ensuring effective treatment. Greater autonomy (µ=4.43) and convenience (µ=4.39) underline that users appreciate being able to treat their condition independently and from home. Motivation to exercise (µ=4.38), cost savings (µ=4.38), and training efficiency (µ=4.37) highlight the comprehensive support offered by the app.

The analysis revealed no statistically significant differences between the perceived benefits of the app and demographic factors or motivation levels. Most respondents stated that they knew the potential benefits of using a mobile app for KOA management. For example, patient 26 said, “The benefits of the app are clear to me, and I think they’re also clear to the others waiting here, regardless of our age or situation. We all know that it can help us manage our condition effectively. That’s why it’s so important. I look forward to using it.”

## Discussion

### Principal Results

This study highlights the importance of motivation, usability, and content quality in adopting app-based therapy for KOA management. The findings indicate that exercise videos, goal setting, and clinician-patient communication are among the most valued app features, emphasizing the need for personalized digital interventions. While motivation levels significantly influenced the perceived usefulness of specific app functionalities, demographic factors such as age, gender, and education played a less prominent role in shaping user preferences.

Barriers to app adoption were primarily related to unclear exercise instructions, inaccurate health information, and limited communication with physiotherapists, suggesting that enhanced instructional clarity and interactive support are essential for improving user engagement. In addition, age-related differences in digital literacy and time constraints highlight the necessity of adaptable app designs that accommodate varying levels of technological proficiency.

The study also reinforces the value of on-demand access to exercise demonstrations, time efficiency, and improved clinician connectivity as key benefits of mobile health interventions. These insights emphasize the need for high-quality exercise content, real-time feedback, and user-friendly interfaces to optimize adherence and clinical effectiveness in digital rehabilitation for patients with KOA.

A correlation analysis between the 2 predominant patient-centric barriers (self-motivation and time) and the motivation measures revealed no significant associations, suggesting that, within our sample’s limitations, these perceived barriers did not directly correspond to participants’ self-reported motivation.

### Comparison With Prior Work

The study revealed several significant findings about using mobile apps for KOA management, aligning with prior research that underscores the accessibility and convenience of app-based therapy for chronic disease management [27,36]. Consistent with previous studies on digital health interventions, our results confirmed that exercise videos were the most highly rated feature, supporting findings that visual demonstrations enhance patient comprehension and adherence to prescribed exercises [21,22,37]. Furthermore, goal setting, tracking, and regular electronic messages were rated highly, reinforcing evidence that structured feedback mechanisms contribute to sustained engagement in mobile health programs [21,27]. The high rating of telephone calls with physiotherapists further highlights the demand for personalized expert guidance, previously identified as a key factor in patient adherence and treatment satisfaction [22,36]. Features that enhance adherence, such as motivational support and automated reminders, were considered essential, echoing findings that digital health interventions incorporating behavioral reinforcement strategies are more effective in promoting long-term compliance [37]. While video calls with physiotherapists and peer communication were rated lower in this study than in some prior research [21,22,37], they remained valuable for individuals seeking real-time interaction or social support, consistent with the findings of Shewchuk et al [36]. This suggests that while many users prefer self-guided app-based therapy, integrating optional interactive elements may enhance engagement for specific subgroups. These results contribute to the growing literature on personalized mobile health interventions, emphasizing the need for flexible, user-centered digital therapy solutions tailored to patient preferences and engagement styles.

Significant differences in the perceived importance of functional requirements were observed based on motivation levels, with highly motivated patients placing greater value on detailed information and practical tools. This finding underscores the need for educational content and visual aids in mobile health apps, consistent with prior research indicating that higher motivation levels correlate with increased engagement in digital health interventions [21,37]. Studies have shown that interactive and visually enriched content enhances patient comprehension and promotes long-term adherence, particularly among those with high intrinsic motivation [22,38]. In addition, opinions on the appropriate duration of physical activity for KOA management also varied significantly by motivation level, reinforcing that individual motivation plays a critical role in adherence to prescribed exercise regimens [21,37]. Research on behavioral interventions for chronic disease management has demonstrated that customizing exercise programs based on motivation levels leads to better adherence and long-term health outcomes [27,39]. Our findings align with this evidence, suggesting that mobile health interventions for KOA should incorporate adaptive exercise planning and motivational support mechanisms to maximize patient engagement and compliance.

Statistically significant gender differences were observed in evaluating the mobile app’s usefulness, with men rating ease of use, clarity of system information, and overall usability higher than women. These findings align with prior research indicating that men prioritize efficiency, simplicity, and functionality in digital health apps. In contrast, women may emphasize engagement, support features, and detail instructional content [24-27,37]. Similar patterns have been reported in health technology adoption studies, where men preferred streamlined, goal-oriented interfaces. At the same time, women were more likely to value interactive elements and comprehensive health-related information [24,25]. These findings underscore the need for gender-sensitive design considerations in mobile health apps, ensuring that user interfaces are adaptable to different preferences and digital literacy levels. Prior studies have emphasized that incorporating customizable settings, adaptive content presentation, and user-driven navigation options can enhance usability and satisfaction across diverse populations [26,27]. Our results contribute to this body of research by reinforcing the importance of personalized digital health solutions that accommodate gender-specific interaction styles, ultimately improving engagement and adherence in app-based therapy for KOA management.

The main barriers to using a mobile app for KOA management identified in this study—inaccurate disease information, unclear exercise presentations, and misunderstanding of information—are consistent with previous research highlighting the critical role of information clarity and accuracy in digital health interventions [25,26,37]. Similar concerns have been reported in telehealth and app-based rehabilitation studies, where patients expressed difficulties in interpreting digital exercise instructions without professional guidance, emphasizing the need for well-structured, evidence-based educational content [36,37]. In addition, difficulties with communication and access to physiotherapists were cited as major challenges, which aligns with prior findings that limited real-time professional interaction can hinder user confidence and adherence to app-based therapy [25,26]. Studies on chronic disease self-management apps have also pointed out that patients often require a balance between self-guided digital tools and clinician oversight. This suggests that incorporating structured communication channels, such as scheduled check-ins or chat-based physiotherapy support, could improve user experience [36]. Significant age-related differences in perceived barriers were observed, reflecting variations in lifestyle demands, digital literacy, and health priorities across different patient groups. Previous research has found that younger individuals prioritize flexibility and convenience, whereas older adults may face more difficulties with app navigation and digital engagement [37]. This underscores the importance of age-sensitive customization, particularly in enhancing information accuracy, integrating time management features, providing motivational support, and ensuring the clarity of exercise instructions. Such adaptations have improved adherence and overall user satisfaction in mobile health interventions for chronic musculoskeletal conditions [25,26,36,37].

Both statistical and qualitative analyses showed that most respondents recognized the potential benefits of using a mobile app for KOA management, regardless of demographic characteristics or motivation levels. This aligns with previous studies highlighting digital health tools’ universal appeal and effectiveness across diverse patient populations [27,37]. Research has shown that mobile health interventions can enhance patient engagement and self-management in chronic conditions, including musculoskeletal disorders, by providing accessible, structured, and adaptable therapy solutions [21,22,36]. Furthermore, studies on app-based therapy for osteoarthritis indicate that perceived usefulness and usability are key determinants of patient adherence, often outweighing differences in age, gender, or baseline motivation [21,37]. Our findings reinforce this notion, suggesting that while individual preferences vary, well-designed digital interventions can effectively support self-management in a broad patient population. This is further supported by evidence indicating that even patients with lower baseline motivation can benefit from mobile health solutions when features such as reminders, structured guidance, and personalized feedback are incorporated into the design [22,27].

### Implications

This study offers several unique contributions to the understanding and developing app-based therapy for KOA management, addressing the gaps not covered in previous research. First, it identifies motivation-specific functional requirements, revealing significant differences in the perceived importance of app features based on motivation levels. Highly motivated patients prioritized features such as detailed symptom tracking and exercise planning, highlighting the need for apps to cater to varying motivation levels to enhance engagement and adherence. Second, the study uncovers gender differences in usability, with men rating ease of use, clarity of system information, and overall usability higher than women, emphasizing the necessity of considering gender-specific preferences in app design. Third, it identifies age-related barriers, noting that younger participants reported time management issues, while older participants were concerned with the accuracy of health information and technical challenges. Tailoring app features to address these age-specific concerns can significantly improve user satisfaction and retention. Fourth, the study finds a universal consensus on exercise duration, with no significant differences in opinions about the appropriate duration of physical activity across gender, age, or education levels, suggesting that general exercise guidelines are broadly applicable. Fifth, it highlights the importance of repeatable instructions, with patients’ highest-rated benefit being the ability to view exercises multiple times, providing a clear direction for developing user-friendly educational materials within the app. Finally, using a mixed methods co-design approach, integrating quantitative and qualitative data, provides a comprehensive understanding of patient needs and preferences, offering a nuanced view of the factors influencing app adherence and satisfaction that single-method studies may miss.

### Limitations

Several limitations of this study should be acknowledged. First, the sample size of 82 patients, while sufficient for preliminary insights, may not represent the broader patient population with KOA. Recruitment from a single orthopedic clinic in Slovenia may limit the generalizability of the findings. Second, the mixed methods approach simultaneously captures patient preferences and barriers, which may not reflect changes over time. Third, reliance on self-reported data introduces potential biases, such as social desirability and recall bias. Although the mixed methods approach enriched the findings, future research could benefit from a longitudinal design to assess the long-term impact of mobile app usage on KOA management outcomes. Fourth, future research should investigate long-term adherence mechanisms and their effects on treatment outcomes to enhance the effectiveness and sustainability of mobile health interventions for KOA management. Fifth, because only 2 participants younger than 40 years were included, the SD in this subgroup was large, limiting the generalizability of our findings for this demographic. Finally, as observed in Slovenia, limited access to physiotherapy and rehabilitation services is a prevalent issue across Eastern and Southern Europe [38,39]. These health care constraints influence the expectations and priorities of patients with KOA for app-based management, highlighting the need for digital solutions tailored to regions with restricted physiotherapy availability. Therefore, addressing these limitations through innovative mobile health interventions is crucial for improving patient outcomes. Future research should validate these findings in diverse health care settings to assess the broader applicability of mobile health interventions.

### Conclusions

This study demonstrates the usefulness of a mixed methods co-design approach for developing mobile apps for KOA management. The findings suggest that app-based therapy for KOA management should prioritize user-centered features such as accessibility, motivation, and clear communication. Smartphones and PCs should be optimized for ease of use with high-quality exercise videos. Integrating motivational features such as goal setting, symptom tracking, and regular e-messages can enhance patient engagement. Personalized communication strategies are crucial, with men preferring straightforward interfaces and all users benefiting from explicit educational content. Addressing barriers such as inaccurate information, unclear instructions, and access issues is essential. Age-specific features should provide flexible scheduling for younger users and detailed support for older users. Ensuring that exercises can be replayed emphasizes the need for clear, repeatable instructions. These considerations can help develop effective, user-friendly app-based therapy that improves KOA management and increases patients’ quality of life, especially in countries with limited access to physiotherapy, such as Slovenia and comparable countries.

## Supplementary material

10.2196/64607 Multimedia Appendix 1 Survey questionnaire.

## Abbreviations

KOA: knee osteoarthritis
