# Supplementary material for: Patients’ Expectations for App-Based Therapy in Knee Osteoarthritis: User-Centered Design Approach
Source: JMIR Rehabil Assist Technol. 2025 May 15;12:e64607. doi: 10.2196/64607 (PMC12097652; doi:10.2196/64607)
Supplement: Multimedia Appendix 1 [file rehab-v12-e64607-s001.docx]

**Demographic data**

| 1. **Gender:**   a) men  b) female  c) I don't want to answer   1. **Completed age:**   (a) up to 40 years  b) 41-50 years  c) 51-60 years  d) 61-70 years  e) 71-80  (f) more than 80 years |  | 1. **Education completed:** 2. Primary 3. Secondary education 4. Higher education 5. Higher education 6. University 7. specialisation or master's degree 8. Doctorate of Science 9. **How much time a day do you usually sit?** 10. I sit minimal (up to 2 hours) 11. Sitting a little (2 to 3 hours) 12. I sit half the day 13. I sit most of the day 14. I sit almost all the time |
| --- | --- | --- |

**Motivation**

1. **On a scale of 1 to 10, please rate your current motivation to regularly perform exercises to increase knee joint flexibility and strengthen your muscles.** (Circle the number that applies most to you)

I am not motivated at all. I am highly motivated.

| 1 | 2 | 3 | 4 | 5 | 6 | 7 | 8 | 9 | 10 |
| --- | --- | --- | --- | --- | --- | --- | --- | --- | --- |

**Expectations for a web app to increase knee joint mobility and strengthen muscles**

1. **The following questions are about what is essential for you to include in a website or mobile app to increase knee joint mobility and muscle strengthening**

(Circle the number that matters most to you: 1− not at all important, 10 extremely important)

| **How important it is to:** | | | | | | | | | | |
| --- | --- | --- | --- | --- | --- | --- | --- | --- | --- | --- |
| 1. the app presents key information on knee arthritis, treatment strategies and recommended lifestyle? | 1 | 2 | 3 | 4 | 5 | 6 | 7 | 8 | 9 | 10 |
| 1. does the app help you set goals to increase knee joint mobility and muscle strengthening, achieve them and measure progress? | 1 | 2 | 3 | 4 | 5 | 6 | 7 | 8 | 9 | 10 |
| 1. the app includes videos with exercises to increase knee joint mobility and strengthen muscles that you could do regularly? | 1 | 2 | 3 | 4 | 5 | 6 | 7 | 8 | 9 | 10 |
| 1. does the app motivate the patient (e.g. email reminder) to do the exercises every week? | 1 | 2 | 3 | 4 | 5 | 6 | 7 | 8 | 9 | 10 |
| 1. the app reminds the patient (email reminder) if they have not logged in to the mobile app to do the exercises for three days? | 1 | 2 | 3 | 4 | 5 | 6 | 7 | 8 | 9 | 10 |
| 1. the app allows the patient to communicate with the physiotherapist by allowing the patient to submit questions via the app? | 1 | 2 | 3 | 4 | 5 | 6 | 7 | 8 | 9 | 10 |
| 1. does the patient using the app can talk to the physiotherapist once a week via phone? | 1 | 2 | 3 | 4 | 5 | 6 | 7 | 8 | 9 | 10 |
| 1. the app allows the patient to communicate with the physiotherapist once a week via video call? | 1 | 2 | 3 | 4 | 5 | 6 | 7 | 8 | 9 | 10 |
| 1. does the app allow patients to communicate with each other? | 1 | 2 | 3 | 4 | 5 | 6 | 7 | 8 | 9 | 10 |

1. **A reasonable amount of time to spend on the app exercises is:**
2. up to 15 minutes
3. 16 min− 30 min
4. 31 min− 45 min
5. **Please indicate how much you agree with the statements regarding the usefulness of the web-based exercise app for increasing knee joint flexibility and strengthening muscles.** (Mark with an X).
6. **Ease of use of the app**

| **Claims** | I don't agree at all | I disagree | I neither agree nor disagree | I agree | I totally agree |
| --- | --- | --- | --- | --- | --- |
| The app must be easy to use. |  |  |  |  |  |
| The information in the app needs to be well organised so I can find the information I need easily. |  |  |  |  |  |

1. **Arranging system information**

| **Claims** | I don't agree at all | I disagree | I neither agree nor disagree | I agree | I totally agree |
| --- | --- | --- | --- | --- | --- |
| The app must allow me to easily and quickly correct any mistakes I make when using the app. |  |  |  |  |  |
| The app must provide an appropriate way to receive instructions for the safe conduct of the exercise. |  |  |  |  |  |
| The app should clearly show how the exercise is going. |  |  |  |  |  |
| Navigation between tabs should be easy. |  |  |  |  |  |

1. **Usability**

| **Claims** | I don't agree at all | I disagree | I neither agree nor disagree | I agree | I totally agree |
| --- | --- | --- | --- | --- | --- |
| The app must include instructions on performing the tested exercises to increase knee joint mobility and strengthen muscles. |  |  |  |  |  |
| The app should help you to do the exercises correctly. |  |  |  |  |  |
| The app should facilitate communication with the physiotherapist. |  |  |  |  |  |
| Using the app, I need several options to interact with the physiotherapist. |  |  |  |  |  |
| I must ensure that any information I send to the physiotherapist via the app will be received. |  |  |  |  |  |

1. **What would be an obstacle to using an online app for knee joint mobility and muscle strengthening exercises** (insert X)**?**

| **Barriers** | **Possible barriers to using the web app** | | | | |
| --- | --- | --- | --- | --- | --- |
|  | I don't agree at all | I disagree | I neither agree nor disagree | I agree | I totally agree |
| A lack of technical knowledge would hinder me from using an online exercise app. |  |  |  |  |  |
| Poor internet access would be a barrier to using an online exercise app. |  |  |  |  |  |
| Inaccurate information about my illness would be a barrier to using an online exercise app. |  |  |  |  |  |
| Not understanding the information would be a barrier to using the online training app. |  |  |  |  |  |
| Lack of time would be a barrier to using the online exercise app. |  |  |  |  |  |
| A lack of self-motivation would hinder me from using an online exercise app. |  |  |  |  |  |
| I would find it difficult to access a physiotherapist via an online app. |  |  |  |  |  |
| I would find it difficult to communicate with the physiotherapist via the web app. |  |  |  |  |  |
| I would consider the unclear presentation of the exercises via the web app to be a barrier. |  |  |  |  |  |
| Inadequate exercise space would be a barrier to using an online exercise app. |  |  |  |  |  |

1. **What would be your advantage in using an online app to exercise to increase knee joint flexibility and strengthen the muscles of** (insert X)**?**

| **Benefits** | **Benefits of using the web app** | | | | |
| --- | --- | --- | --- | --- | --- |
|  | I don't agree at all | I disagree | I neither agree nor disagree | I agree | I totally agree |
| The time savings would be an advantage for me when using an online exercise app. |  |  |  |  |  |
| Saving money would be an advantage for me when using the web app. |  |  |  |  |  |
| More autonomy would be an advantage for me when using the app. |  |  |  |  |  |
| I would see being more motivated when using the app as an advantage. |  |  |  |  |  |
| I would find it more convenient and advantageous to use the web app. |  |  |  |  |  |
| I benefit from easier access to a physiotherapist. |  |  |  |  |  |
| Easier communication with the physiotherapist would be an advantage when using the web app. |  |  |  |  |  |
| I would consider the accuracy/veracity of the information to be an advantage. |  |  |  |  |  |
| I would consider it an advantage to use it more efficiently. |  |  |  |  |  |
| I would consider the possibility of viewing the exercises multiple times to be an advantage when using the web app. |  |  |  |  |  |

***Thank you for your participation!***
